# Mobile Payment Protocol with Deniably Authenticated Property

**DOI:** 10.3390/s23083927

**Published:** 2023-04-12

**Authors:** Yunzhuo Liu, Wen Huang, Ming Zhuo, Shijie Zhou, Mengshi Li

**Affiliations:** 1 School of Information and Software Engineering, University of Electronic Science and Technology of China, Chengdu 610054, China; 2 Colleague of Computer Science, Sichuan University, Chengdu 610017, China

**Keywords:** deniable authentication, mobile payment, deniably authenticated encryption, privacy preserving, confidentiality

## Abstract

Mobile payment services have been widely applied in our daily life, where users can conduct transactions in a convenient way. However, critical privacy concerns have arisen. Specifically, a risk of participating in a transaction is the disclosure of personal privacy. This might occur if, for example, the user pays for some special medicine, such as AIDS medicine or contraceptives. In this paper, we propose a mobile payment protocol that is suitable for mobile devices only with limited computing resources. In particular, the user in a transaction can confirm the identity of others in the same transaction while the user cannot show convincing evidence to prove that others also take part in the same transactions. We implement the proposed protocol and test its computation overhead. The experiment results corroborate that the proposed protocol is suitable for mobile devices with limited computing resources.

## 1. Introduction

In recent years, mobile communication technology has developed rapidly, especially in rural areas. In the past, due to the lack of infrastructure for communication, people in rural areas did not have easy access to the Internet. With the application of mobile communication devices such as 5G facilities, people in rural areas are becoming increasingly connected to the world. For example, more and more people are shopping via mobile communication networks.

With the popularity of mobile devices and the increasing dependence of people’s lives on mobile devices, mobile payment has become widely used around the world. Many organizations, for example, banks, software companies, and mobile operators, have already made many efforts to promote mobile payment services. Google Wallet, MasterPass, Android Pay, and Apple Pay are well-known services in the mobile payment market. One study named Transparency Market Research [1] shows that the market of global mobile payment will reach USD 1602.4 billion.

Although the widespread use of mobile payment services has brought great convenience to people, daunting privacy challenges related to the disclosure of transaction information have arisen, and limited computing resources of mobile devices have also become a problem in the application of mobile payment services. In particular, the private information involved in a transaction brings the risk of disclosure of personal privacy. For example, the US-based mobile social payment platform named Venmo generates payment notes for each Venmo transaction, and these payment notes are visible to all other Venmo users. Rajat et al. found that “41 M notes (10.5%) leak some sensitive information such as health condition, political orientation and drug/alcohol consumption involving 8.5 M (37.8%) users” [2]. In some cases, the risk of participating in a transaction is the disclosure of personal privacy. This might occur if, for example, the user pays for some special medicine, such as AIDS medicine or contraceptives.

To safeguard personal privacy, entities in some application scenarios might not want others to know that they have taken part in a transaction. In a word, it is critical in the circumstances described above that parties in a transaction cannot convince others to believe that a particular person takes part in the transaction.

A deniably authenticated encryption scheme can ensure that it is possible to confirm the identity of entities in the transaction, while also ensuring that there is no chance to affirmatively show the identity of entities in the transaction to other parties [3]. In addition, mobile payment services run on mobile devices with limited computing resources, so there is a need to a design a lightweight mobile payment protocol. Thus, we utilize the deniably authenticated encryption method to construct a mobile payment protocol that can protect the privacy of the client involved in a transaction.

### 1.1. Contribution

We design a mobile payment protocol that is suitable for application in a mobile payment scenario, where privacy preservation is critical. Firstly, the designed protocol is lightweight. In particular, we adopt an optimized deniably authenticated encryption scheme that can be applied on mobile devices with limited computing resources. Secondly, the proposed protocol can satisfy multiple design goals at the same time. Specifically, the designed protocol has the properties of confidentiality, integrity, deniable authentication, traceability, and non-repudiation.

### 1.2. Related Work

The discussion about the related works involves two aspects: deniable authentication and mobile payment.

Authentication ensures that the identity of the communicating entity is in line with its own claims. It is a security feature that is necessary in many application scenarios, for instance, smart meters [4], the Metaverse [5], and the location authentication of mobile devices [6,7].

In some application scenarios, an authentication scheme with deniability is needed. Deniable authentication guarantees two particular features. First, it is possible to confirm the identity of the entities in the transaction. Second, there is no chance of showing the identity of entities in the transaction to other entities affirmatively.

On the basis of their original work relating to zero-knowledge proof, Dwork et al. developed a deniable authentication protocol [8]. However, their protocol was limited by a timing constraint called the (α,β) assumption on the response time of processes.

Aumann and Rabin devised a deniable authentication method based on the factoring issue, a tough topic in computing complexity theory [9]. Deng et al. developed a new deniable authentication strategy based on a tough problem in computation complexity theory known as discrete logarithm and factoring problems [10]. The methods of [9,10] assumed that there was a trustworthy third entity who was able to provide a public directory service. On the basis of [11], Fan et al. [12] developed a deniable authentication protocol to improve the protocols of [9,10]. Public key certificates on their protocol prevented person-in-the-middle (PIM) attacks, and their protocol verified the source of messages using digital signatures. However, Yoon et al. [13] proved that [12] is vulnerable regarding authentication. An attacker was able to disguise as the receiver and confirm the identity of the sender.

Based on the ElGamal signature scheme [14], Shao [15] built a deniable authentication scheme over which involved entities did not have to interact with each other. In 2005, Lu developed a deniable authentication method based on a tough problem in computation complexity theory known as the factoring problem, in which involved entities did not have to communicate with each other [16]. The security of their protocol was shown in the random oracle model. Wang [17] created a deniable authentication protocol based on the ElGamal cryptosystem [18] with the inverse of the ElGamal implementing deniable authentication. According to Shao [19], the protocol of [17] has a weakness. An attacker can use this flaw to launch the PIM attack. The attacker can impersonate a legitimate communication entity. Yoon et al. [20] suggested in 2010 that Shao [19]’s enhanced protocol is not safe against a receiver impersonation attack.

Based on ElGamal cryptography, Yoon et al. proposed an improved deniable authentication protocol to overcome this weakness [20]. However, Li and Takagi argued in [21] that [20] did not have the deniable authentication property. The receiver can reveal the sender’s identify to other entities. Based on the ElGamal signature scheme [14], a deniable authentication procedure was presented by Lee et al. [22]. Even if the signature mechanism was compromised, the protocol can operate as a signature scheme to provide security.

Based on the DDH assumption, Wang et al. developed a deniable authentication procedure in which participants did not need to engage with one another [23]. Li et al. built a new deniable authentication protocol that used an identity-based cryptology system [24]. Liao et al. developed a system that used a secure certificateless signing mechanism and delegated signature verification to a cloud server [25]. Li et al. presented a technique that can accomplish deniable authentication, confidentiality, and integrity [26]. After Li’s scheme, many other deniably authenticated schemes were proposed, such as [27,28,29,30].

Researchers from home and abroad have given the definition of mobile payment, which is as follows: a mobile payment is a form of payment transaction processing over mobile communication techniques in which the payer initializes, authenticates, and completes payment by mobile devices [31]. Thus far, there have been some different proposed mobile payment protocols, such as [32,33]. These protocols are based on symmetric cryptography. These protocols are efficient and suitable for mobile devices. However, these protocols have various weaknesses. Other protocols using public key cryptography are not appropriate for mobile devices because they are designed for fixed networks, for instance. In addition, privacy preservation is also an important requirement of mobile payment protocols. Therefore, in this paper, we optimize a deniably authenticated encryption scheme, making it suitable for mobile devices, and then we apply it to mobile payment protocols.

The concept of deniably authenticated encryption was created by Li et al. [26]. It has been applied to many fields. Based on identity cryptography, Chunhua et al. improved the communication costs and ciphertext size of deniably authenticated encryption schemes [34]. Managing certificates costs a lot [35,36,37], and their scheme can avoid public-key-certificate-based infrastructure. Kasper et al. named the concept of strong and weak deniable authentication and then proposed two efficient encryption schemes that provide deniable authentication [38]. To protect the privacy of location data, Guanhua et al. proposed a deniable authenticated encryption scheme based on certificateless cryptosystems [39]. Their scheme did not depend on public key infrastructure and avoided the problem of key escrow present in identity-based cryptosystems. Chunhua et al. proposed a deniable authentication encryption scheme based on an identity-based environment to avoid public-key-certificate-based public key infrastructure, and then they applied their scheme into the scenario of e-voting. Ahene et al. also constructed an e-voting system based on a deniably authenticated encryption scheme [28]. Kar et al. applied deniably authenticated encryption into the field of e-mail [29]. Wen et al. protected the privacy of entities in communications through a deniably authenticated encryption scheme [40]. Based on bilinear pairings, Chunhua et al. constructed a deniably authenticated encryption for e-mail applications [41]. Chunhua et al. proposed the concept of heterogeneous deniable authenticated encryption, which enabled a sender in a public key infrastructure environment to transmit a message to a receiver in an identity-based environment [42]. Based on blockchain and deniably authenticated encryption technology, Zhang proposed a deniably authenticated searchable encryption scheme to guarantee the privacy and workability of medical image data [30]. Jin et al. used a deniably authenticated encryption scheme to implement location-based services [43]. Based on a two-user ring signature, Shengke et al. constructed an efficient deniable authentication scheme to protect location privacy [44]. Yanmei et al. formalized the syntax and security notions of public-key-authenticated deniable encryption, and then proposed two concrete constructions under a fully deniable framework [45].

### 1.3. Organization

In the next section, we introduce preliminaries of deniably authenticated encryption. In Section 3, we elaborate on the system model and design goals. In Section 4, the proposed protocol is presented in detail. In Section 5, the security analysis of the proposed protocol is given. In Section 6, we theoretically analyze the computational complexity, and then we implement the proposed protocol and test running time of our implementation. Finally, we provide our conclusion.

## 2. Preliminaries

### 2.1. Deniably Authenticated Encryption

The concept of deniably authenticated encryption was proposed by Li et al. for a secure e-mail service [26]. It achieves confidentiality, integrity, and deniable authentication in a logical single step. Then, Wen et al. optimized the deniably authenticated encryption scheme [40]. The deniably authenticated encryption scheme consists of four algorithms, including the setup, keygen, DA-encrypt, and DA-decrypt algorithms.

**Setup.** The Setup algorithm takes the security parameter λ as input and generates system parameters.

**KeyGen.** The KeyGen algorithm produces keys for the sender and receiver. In particular, it generates a public key $y_{s}$ and a private key $x_{s}$ for the sender, and it generates a public key $y_{r}$ and a private key $x_{r}$ for the receiver.

**DA-Encrypt.** The sender produces ciphertext σ of message *m* by performing DA-Encrypt algorithm. Specifically, the DA-Encrypt takes system parameters generated by Setup algorithm, message *m*, sender’s private key $x_{s}$, sender’s public key $y_{s}$ together with receiver’s public key as inputs, and then produces ciphertext σ of message *m*.

**DA-Decrypt.** The receiver can extract plaintext *m* from a ciphertext σ through the DA-Decrypt algorithm. More specifically, it takes the system parameters, ciphertext σ, receiver’s private key $x_{s}$, receiver’s public key $y_{s}$, and the sender’s public key as inputs, and then outputs the plaintext *m* of a ciphertext σ if the ciphertext σ is a valid ciphertext. DA-Decrypt outputs nothing if ciphertext σ is an invalid ciphertext.

### 2.2. Security Concepts

**Definition** **1.** *A deniable authenticated encryption scheme is $\left(\epsilon_{dea},t,q_{e},q_{d}\right)$-IND-CCA secure if there is no adversary A who has an advantage of at least $\epsilon_{dea}$ in the IND-CCA game under the condition that time is probabilistic t-polynomial time, that deniably authenticated encryption queries are, at most, $q_{e}$, and that deniably authenticated decryption queries are, at most, $q_{d}$.*

**Definition** **2.** *A deniable authenticated encryption scheme is $\left(\epsilon_{dea},t,q_{e},q_{d}\right)$-DA-CMA secure if there is no adversary A who has an advantage of at least $\epsilon_{dea}$ in the IND-CCA game under the condition that time is probabilistic t-polynomial time, that deniably authenticated encryption queries are, at most, $q_{e}$, and that deniably authenticated decryption queries are, at most, $q_{d}$.*

Definition 1 as well as Definition 2 are from [40], and the detailed information of the IND-CCA game is elaborated in [40].

## 3. System Model

**Figure 1:**
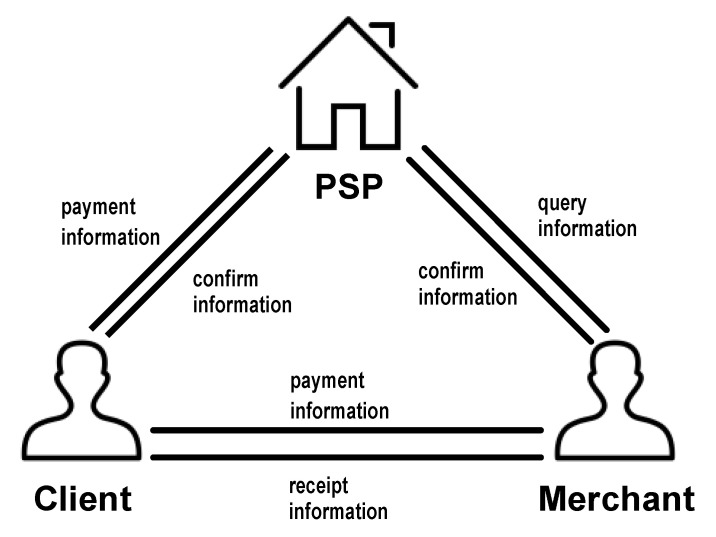
System model.

The model of payment systems is demonstrated in Figure 1. A client, a merchant, and a payment service provider (PSP) make up this model. The client, Alice, is the entity who wants to pay for the merchant. The merchant, Bob, is the entity who receives money from someone else, such as Alice. PSP Bank is a financial infrastructure that provides payment services while also storing the account information of the entities involved. We assume that the bank is a trusted entity and it never does anything evil.

### 3.1. Threat Model

In our protocol, we assume that the attack can control the client or the merchant. When the attacker controls the client, his goal is to deny his participant in a transaction so that he can obtain products without non-payment. When the attacker controls the merchant, his goal is to convince others to believe that the client takes part in a transaction. The exposure of purchase records may leak the privacy information of the client, especially the records related to special products, such as special medicines. The merchant may make unjustified profits by selling the client’s purchase records when he can convince others to believe that his purchase records are true.

### 3.2. Design Goal

According to the requirements of the application scenario, the secure mobile payment protocol should satisfy properties of confidentiality, deniable authentication, integrity, traceability, non-repudiation, and small overhead.

**Confidentiality:** assures that only the intended recipient is aware of the message’s contents.

**Integrity:** ensures that messages sent during the interaction process are not tampered with by unlawful entities.

**Deniable authentication:** ensures that the entity receiving the message can confirm the identity of the entity sending it. Furthermore, the entity receiving the message is unable to reveal the identity of the entity sending the message to the third entity.

**Traceability:** ensures that the client cannot deny that she made the payment herself. Meanwhile, the merchant cannot deny the received payment. Otherwise, the unique identity stored in PSP can be used to track them.

**Non-repudiation:** once a merchant confirms the information message, he or she cannot deny the communication’s validity or origin. Additionally, once a client acknowledges the information message, he or she is unable to retract her confirmed payment message.

**Lightweight overhead:** because the mobile payment protocol is used on mobile devices with limited computing resources, the mobile payment protocol needs to be lightweight.

## 4. Proposed Protocol

**Figure 2:**
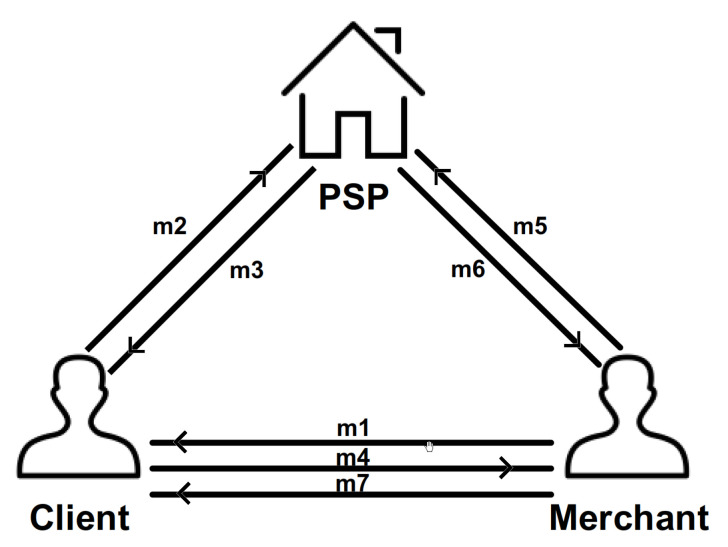
Proposed Protocol.

The proposed protocol consists of three phases, including the setup phase, keygen phase, and payment transaction phase. In the setup phase, PSP obtains security parameters λ and outputs system parameters. In the keygen phase, the client obtains the system parameters and outputs the account and the code of the account. Next, the client sends his or her ID and account to PSP. Then, the merchant performs all the same activities as the client. In the payment transaction phase, the merchant and the client interact with each other to transfer the payment. The interaction scenario among the above entities is sketched in Figure 2. Unlike Figure 1 (which elaborates on the components of the whole system), Figure 2 elaborates on how one component interacts with other components in detail.

Next, we give detailed information about the message in the interaction scenario in Figure 2.

$$m_{1}=\left(T_{id}\|Amount_{t}\|ID_{b}\|Timestamping\right)$$

is the payment information, including the ID of the transaction, the amount of the transaction, the ID of the merchant, and the time stamp.

$$m_{2}=\left(T_{id}\|Amount_{t}\|ID_{b}\|Timestamping\|ID_{a}\|Transfer_{a}\right)$$

is the payment message sent from the client to PSP.

$$m_{3}=\left(T_{id}\|Amount_{t}\|ID_{b}\|Timestamping\|ID_{a}\|Transfer_{p}\right)$$

is the payment confirmation message sent from PSP to the client.

$$m_{4}=\left(T_{id}\|Amount_{t}\|ID_{b}\|Timestamping\|ID_{a}\|Confirm_{a}\right)$$

is the confirmatory payment message sent from the client to the merchant.

$$m_{5}=\left(T_{id}\|Amount_{t}\|ID_{b}\|Timestamping\|ID_{a}\|Confirm_{a}\right)$$

is the query message sent from the merchant or client to PSP.

$$m_{6}=yes\;or\;m_{6}=no$$

is the message of response to the query message sent from PSP to the merchant or the client.

$$m_{7}=(T_{id}\|Amount_{t}\|ID_{b}\|Timestammping\|ID_{a}\|Confirm_{b})$$

is the confirmatory receipt message sent from the merchant to the client.

### Implementation of Mobile Payment Protocol

Next, we give a detailed implement of the proposed security mobile payment protocol. As mentioned above, the secure mobile payment protocol consists of three phases.

**Setup Phase:** Initially, PSP obtains the security parameter λ, runs the setup algorithms of the deniable authenticated encryption scheme [40], and outputs the system parameters $\left\{n,p,q,g,H_{2},H_{1}\right\}$. Here, $H_{2}$ and $H_{1}$ are two hash functions that are randomly selected from the system.

**Keygen Phase:** PSP runs the keygen algorithm of the deniable authenticated encryption scheme. The client Alice randomly chooses their private key $x_{a}$ from $Z_{q}^{*}$, computes $y_{a}=g^{x_{a}}\;\mathrm{mod}\;p$, and takes $\left(x_{a},y_{a}\right)$ as its private/public key pair. The client Alice sends its unique $ID_{a}$ and $y_{a}$ to PSP, and then PSP stores them in secure storage. Similarly, the merchant Bob randomly chooses their private key $x_{b}$ from $Z_{q}^{*}$, computes $y_{b}=g^{x_{b}}\;\mathrm{mod}\;p$, and takes $\left(x_{b},y_{b}\right)$ as its private/public key pair. The merchant Bob sends its unique $ID_{b}$ and $y_{b}$ to PSP, and then PSP stores them in secure storage. PSP randomly chooses its private key $x_{p}$ from $Z_{q}^{*}$, computes $y_{p}=g^{x_{p}}modp$, and takes ($x_{p}$,$y_{p}$) as its private/public key pair.

**Payment Transaction Phase:**

➀ Bob constructs his payment request on his website. The payment request consists of the transaction identity, $T_{id}$; the amount to be paid, $Amount_{t}$; his unique identity, $ID_{b}$; and the transaction time stamp, Timestamping.

$$m_{1}=(T_{id}\|Amount_{t}\|ID_{b}\left\|Timestamping\right)$$

Here, ‖ represents the message concatenation.

With the given message m, the key pair ($x_{b}$, $y_{b}$) from Bob, and the public key $y_{a}$ from Alice, Bob encrypts *m* into a ciphertext, $\sigma_{1}$, using the deniably authenticated encryption algorithm. Bob sends $\sigma_{1}$ to Alice.

➁ Alice obtains the ciphertext $\sigma_{1}$. With the ciphertext $\sigma_{1}$, the public key $y_{b}$ from Bob, and the key pair $\left(y_{a},x_{a}\right)$ from Alice, Alice decrypts $\sigma_{1}$ using the deniably authenticated decryption algorithm into $m_{1}$. If proven valid, Alice constructs her transfer confirmation message

$$m_{2}=(T_{id}\|Amount_{t}\|ID_{b}\|Timestamping\|ID_{a}\left\|Transfer_{a}\right)$$

Alice encrypts $m_{2}$ to $\sigma_{2}$ using the deniably authenticated encryption algorithm and then sends $\sigma_{2}$ to PSP.

➂ PSP obtains the ciphertext $\sigma_{2}$. With the PSP’s key pair $(y_{P}$ and $x_{P})$ and Alice’s public key $y_{a}$, PSP decrypts $\sigma_{2}$ into $m_{2}$ using the deniably authenticated decryption algorithm. If $\sigma_{2}$ is valid, PSP stores the content decrypted from the received ciphertext $\sigma_{2}$ in the local database, and PSP constructs its transfer confirmation message $m_{3}$.

$$m_{3}=(T_{id}\|Amount_{t}\|ID_{b}\|Timestamping\|ID_{a}\left\|Transfer_{p}\right)$$

PSP encrypts $m_{3}$ into $\sigma_{3}$ using the deniably authenticated encryption algorithm, then sends $\sigma_{3}$ to Alice.

➃ Alice obtains the ciphertext $\sigma_{3}$. With the PSP’s public key $y_{P}$ and Alice’s key pair $\left(y_{a},x_{a}\right)$, Alice decrypts $\sigma_{3}$ into $m_{3}$ using the deniably authenticated decryption algorithm. If $\sigma_{3}$ is valid, Alice constructs her confirmation message $m_{4}$,

$$m_{4}=(T_{id}||Amount_{t}||ID_{b}\|Timestamping\|ID_{a}\left\|Confirm_{a}\right)$$

Alice encrypts $m_{4}$ into $\sigma_{4}$ using the deniably authenticated encryption algorithm, then sends $\sigma_{4}$ to Bob.

➄ Bob obtains the ciphertext $\sigma_{4}$. With Alice’s public key $y_{a}$ and Bob’s key pair $\left(x_{b},y_{b}\right)$, Bob decrypts $\sigma_{4}$ into $m_{4}$ using the deniably authenticated decryption algorithm. If $\sigma_{4}$ is valid, Bob constructs his query message $m_{5}$

$$m_{5}=(T_{id}||Amount_{t}\|ID_{b}\|Timestamping\|ID_{a}\left\|Confirm_{a}\right)$$

Bob encrypts $m_{5}$ into $\sigma_{5}$ using the deniably authenticated encryption algorithm and then sends $\sigma_{5}$ to PSP.

➅ PSP obtains the ciphertext $\sigma_{5}$. With PSP’s key pair $\left(y_{P},x_{P}\right)$ and Bob’s public key $y_{b}$, PSP decrypts $\sigma_{5}$ using the deniably authenticated decryption algorithm into $m_{5}$. If $\sigma_{5}$ is valid, we must next check whether there is a message $m_{5}$ in the database. If $m_{5}$ is found, PSP constructs a response message, where $m_{6}=yes$ or $m_{6}=no$. PSP encrypts $m_{6}$ into $\sigma_{6}$ using deniably authenticated encryption, then sends $\sigma_{6}$ to Bob.

➆ Bob obtains the ciphertext $\sigma_{6}$. With Bob’s key pair $\left(x_{b},y_{b}\right)$ and PSP’s public key $y_{P}$, Bob decrypts $\sigma_{6}$ into $m_{6}$ using the deniably authenticated decryption algorithm. If $\sigma_{6}$ is valid, Bob constructs his confirmation message $m_{7}$,

$$m_{7}=(T_{id}\|Amount_{t}\|ID_{b}\|Timestamping\|ID_{a}\left\|Confirm_{b}\right)$$

Bob encrypts $m_{7}$ to $\sigma_{7}$ using the deniably authenticated encryption algorithm, then sends $\sigma_{7}$ to Alice.

## 5. Security Analysis

Considering our design goals, we outline how these goals can be achieved using our proposed protocol.

The merchant cannot convince others that the client took part in a particular transaction because the merchant has the ability to forge transaction information.

**Theorem** **1.** *The merchant can forge the legal ciphertext of the client without the private key of the client.*

**Proof.**  Assume that symbol $sk_{a}$ represents the private key of the client and symbol $sk_{a}$ represents the private key of the merchant. There is a message *m*, and the merchant forges their ciphertext as follows:

1. Choose a random number *x* from $Z_{q}^{*}$;
2. Compute $w=y_{b}^{x}\;mod\;p$ and $k=H_{1}\left(w\right)$;
3. Compute c=m⊕k;
4. Compute $e=H_{2}\left(m|\right|y_{b}\left|\right|y_{a}\left||w\right)$. Here, || represents the message concatenation;
5. Compute $v=sk_{a}e+x\;mod\;q$;
6. Compute $z=y_{b}^{v}\;mod\;p$;
7. The forged ciphertext is σ=(c,e,z);.

In the above procedure, $y_{b}$ is the public key of the merchant, $y_{a}$ is the public key of the client; and $H_{1}$ and $H_{2}$ are two hash functions. □

Due to the merchant’s ability to forge the legal ciphertext of the client, the merchant cannot make others believe his claim that some ciphertexts of transaction messages are generated by the client. That is, the client has the ability to deny participation in one transaction, in which it does not matter whether the client took part.

Confidentiality, integrity, and deniable authentication are achieved because the content of the message is encrypted by the deniable authenticated encryption algorithm. Detailed information can be found in Theorem 1 and Theorem 2 in [40].

**Traceability:** the transaction can be tracked by PSP if necessary because the confirmatory message between the client and PSP includes the ID of the client. For example, the court can ask to trace the transaction. Notably, PSP is assumed to be trusted. Thus, we can believe in PSP if it shows evidence that someone participated in a transaction. PSP ensures that it will not show evidence of whether someone participated in a transaction except when the requester has access rights, such as the police, court staff, and so on.

**Non-repudiation:** the confirmatory message between the client and PSP includes the ID of the client, and we trust that PSP does not do anything evil. Therefore, the client is not able to repudiate the correctness and the origin of the message once he or she confirms the message. Similarly, the merchant cannot deny his or her confirmatory message if he or she did send the confirmatory message.

## 6. Efficiency Comparison and Verification

For the simplicity of description, we use the below symbols. The symbol ✓ denotes that the proposed protocol has corresponding characteristics. The length of the message is represented by |χ|. The times for modular inverse, modular multiplication, modular exponentiation, and hash function operations are represented by $T_{i},T_{m},T_{e}$, and $T_{h}$, respectively. The theoretical analysis of the computational complexity of the proposed protocol is shown in Table 1.

Next, we took the running time as the standard to quantify computational complexity. The running time is dependent on the equipment on which experiments are performed, so we first introduced our experiment environment. We implemented the proposed protocol on an Ubuntu 12.04 virtual machine, which was installed on an Intel Core i5 2.6GHz computer with 8G RAM, using the MIRACL library [26]. When it comes to implementing huge-number cryptography, the MIRACL library is commonly regarded as a useful building kit for creating cryptography systems. Thus, we chose MIRACL to implement the proposed protocol.

**Figure 3:**
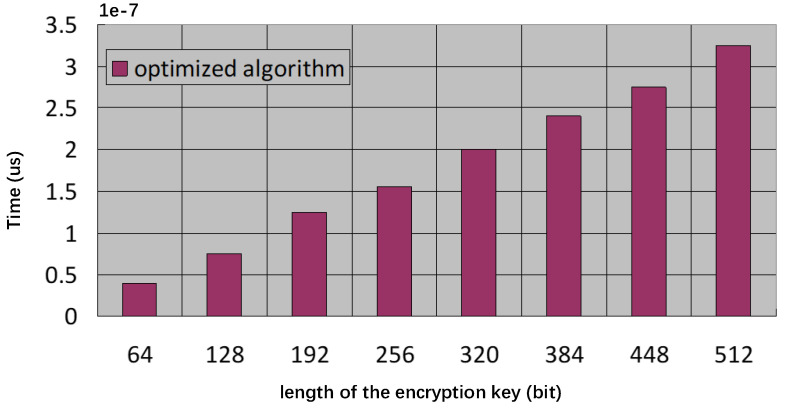
Encryption Time Comparison.

In the first experiment, we tested the running time(computational complexity) of the encryption operation. In particular, *p* is 1024 bits and *q* is 512 bits in the proposed protocol. We employed eight different encryption key lengths, starting with 64 bits and gradually increasing this number by 64 bits. At an eight-type encryption key length, Figure 3 shows the running time (sum time to execute 1000 iterations of deniably authenticated encryption).

**Figure 4:**
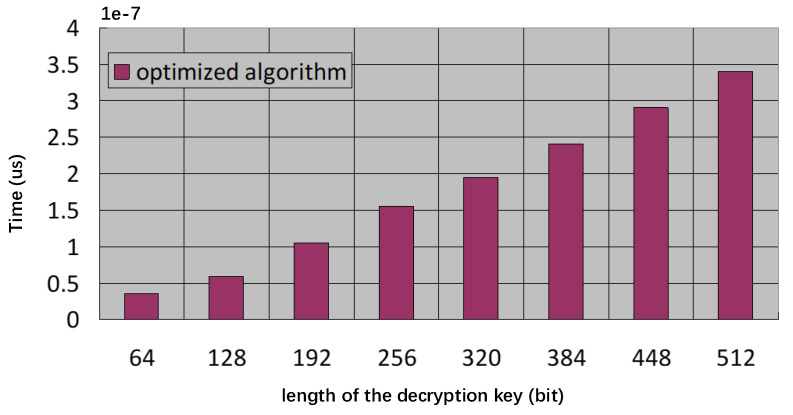
Decryption Time Comparison.

In the second experiment, we tested the running time of the decryption operation. Specifically, we chose 1024 as the value of *p*, and we chose 512 as the value of *q*. We employed eight different decryption key lengths, starting with 64 bits and gradually increasing this number by 64 bits. The experiment results are demonstrated in Figure 4. According to the experimental results, the proposed protocol is lightweight; that is, the proposed protocol can be applied into the application scenario of mobile payments.

## 7. Conclusions

In order to preserve the privacy of entities of particular transactions, we designed a mobile payment protocol that can be applied into mobile devices, such as a POS machine, with limited computing resources. The entity in a transaction can confirm the identity of other entities involved in the transaction, and the entity in a transaction cannot show convincing evidence to prove that other entities are involved in the transaction. We implement the proposed protocol by an optimized deniably authenticated encryption, making it suitable for mobile devices.

## Figures and Tables

**Table 1:** Performance analysis.

Computational Cost		Ciphertext Size	Security		Formal Proof	No-Interactive
Sender	Receiver		NID-CCA	DA-CMA		
$2T_{h}$+ $2T_{e}+T_{m}$	$2T_{h}$+ $2T_{e}+T_{m}$	|m|+ |q|+|p|	√	√	√	√

## Data Availability

Not applicable.
